# Sensitivity of circulating tumor DNA in advanced mycosis fungoides: A retrospective case series

**DOI:** 10.1016/j.jdin.2026.05.016

**Published:** 2026-05-25

**Authors:** Jennifer DeSimone, Nidhi Kuchimanchi, Jafar Al-Mondhiry, Sekwon Jang

**Affiliations:** a Inova Schar Cancer Institute, Fairfax, Virginia; b University of Virginia School of Medicine, Charlottesville, Virginia

**Keywords:** advanced mycosis fungoides, biomarker, circulating tumor DNA, CTCL, ctDNA, sensitivity

*To the Editor:* Circulating tumor DNA (ctDNA) is a personalized biomarker which quantifies tumor-derived DNA shed into the bloodstream and has demonstrated prognostic and surveillance value in melanoma, Merkel cell carcinoma, and systemic lymphomas.^1^, ^2^, ^3^ However, its utility in cutaneous T-cell lymphoma, particularly advanced mycosis fungoides (MF), remains largely unexplored. We evaluated the baseline sensitivity of tumor-informed Signatera ctDNA in patients with advanced MF.

This retrospective case series was conducted at a single academic center and included all consecutive patients with at least Stage IIB MF and more than 4 tumors encountered in clinic between January 2025 and December 2025, all of whom underwent baseline tumor-informed Signatera ctDNA testing. All patients had active cutaneous disease at the time of ctDNA testing. The primary end point was sensitivity of detectable ctDNA (≥0.01 MTM/mL) at baseline. Serial measurements were obtained at 6-8 week intervals as part of routine care.

Twelve consecutive patients with Stage IIB-IVB MF underwent ctDNA testing and all had detectable ctDNA levels (100%, Fig 1). The cohort included 5 male and 7 female patients with a median age of 62 years (range 35-85). Five patients had Stage IIB disease (41.67%), 5 patients had Stage IVA disease (41.67%), and 2 patients had Stage IVB disease (16.67%). Seven patients had large cell transformation (58.33%, Table I). Baseline values ranged from 0.06 to 2092.03 MTM/mL. The median value was 58.64 MTM/mL. An outlier value of 2092.03 MTM/mL was detected in a patient with extremely bulky tumors and visceral metastatic large cell transformed disease. The median value omitting this outlier was 45.38 MTM/mL. The number of subsequent ctDNA values for disease tracking ranged from 1 to 15 with the longest duration of follow up being 20 months.

**Fig 1:**
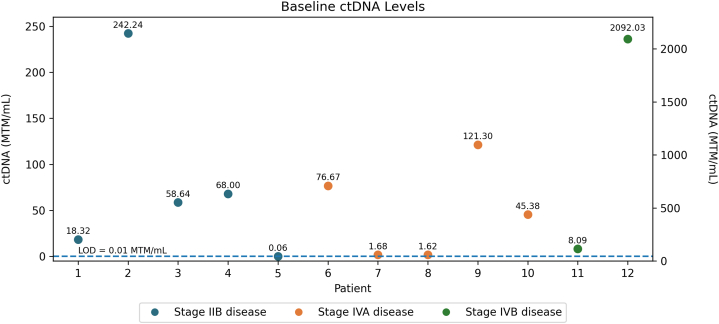
Advanced mycosis fungoides. Scatter plot of ctDNA values for patients 1-12, showing 100% sensitivity. *ctDNA*, Circulating tumor DNA.

**Table I:** Advanced mycosis fungoides

Demographics and disease characteristics of 12 patients with advanced mycosis fungoides	
Sex	
Male	5/12 (41.67%)
Female	7/12 (58.33%)
Age	
31-40 y	1/12 (8.33%)
41-50 y	1/12 (8.33%)
51-60 y	3/12 (25.00%)
61-70 y	3/12 (25.00%)
71-80 y	2/12 (16.67%)
81-90 y	2/12 (16.67%)
Disease stage	
I	0/12 (0%)
II	
IIA	0/12 (0%)
IIB	5/12 (41.67%)
III	0/12 (0%)
IV	
IVA	5/12 (41.67%)
IVB	2/12 (16.67%)
Presence of large cell transformation?	
Yes	7/12 (58.33%)
No	5/12 (41.67%)
Current therapy	
Brentuximab	5/12 (41.67%)
Brentuximab + Electron beam radiation	1/12 (8.33%)
Liposomal doxorubicin	3/12 (25.00%)
Liposomal doxorubicin + Electron beam radiation	1/12 (8.33%)
Ruxolitinib	1/12 (8.33%)
Pembrolizumab	1/12 (8.33%)

Advanced MF is molecularly heterogeneous, and no universal biomarkers currently exist. It is chronic and incurable, and durable complete responses are rare. Assessments are primarily based on clinical exam, which in the advanced setting typically includes some level of smoldering disease. Patients often display discordant activity in various skin lesions, making determination of early treatment response highly nuanced and potentially inaccurate. Similarly, progressive and recurrent disease is difficult to decipher, which impacts optimal retreatment timing. A blood biomarker able to gauge treatment response and MRD is needed.

In this cohort of patients with advanced MF, ctDNA was detectable in all cases, suggesting high baseline sensitivity of a tumor-informed assay in this disease. Testing was applied to patients with tumor-stage disease and high cutaneous tumor burden, which may limit generalizability. Serial measurements are ongoing to evaluate the potential role of ctDNA in longitudinal disease monitoring and predictive assessment. These findings highlight ctDNA as a promising adjunctive biomarker in MF, a disease where clinical assessments are variable and objective laboratory markers are lacking. Additional limitations include the small sample size, retrospective single-center design, and heterogeneity of systemic therapies. Prospective studies are needed to determine whether ctDNA can guide treatment decisions or serve as a measure of minimal residual disease in cutaneous T-cell lymphoma.

## Conflicts of interest

None disclosed.
